# Predicting long-term outcomes in intracerebral hemorrhage: a comparative study of machine learning models highlights the prognostic value of hematoma clearance

**DOI:** 10.3389/fneur.2026.1828200

**Published:** 2026-08-28

**Authors:** Cheng Zhang, Yuchen Jiang, Jiaqi Lin, Shengsheng Ge, Zhonghuai Zhang, Ling Yuan, Hangzhe Sun

**Affiliations:** 1 Department of Neurosurgery, Shengzhou Traditional Chinese Medicine Hospital, Shengzhou, Zhejiang, China; 2 Second Clinical Medical College, Zhejiang University of Chinese Medicine, Hangzhou, China; 3 The First Clinical Medical College, Wenzhou Medical University, Wenzhou, Zhejiang, China; 4 School of Pharmacy, Hainan Medical University, Haikou, Hainan, China

**Keywords:** hematoma clearance, intracerebral hemorrhage, logistic regression, long-term outcome, machine learning

## Abstract

**Purpose:**

This study aimed to develop and internally validate postoperative prognostic models for long-term functional outcomes in patients with spontaneous intracerebral hemorrhage (sICH) following hematoma evacuation, and to compare the performance of traditional logistic regression (LR) with machine learning (ML) algorithms in a limited sample size setting.

**Methods:**

A retrospective cohort of 185 ICH patients who underwent stereotactic surgery was analyzed. Four key predictors—age, admission Glasgow Coma Scale (GCS) score, ICH grade, and hematoma clearance ratio—were selected using Least Absolute Shrinkage and Selection Operator (LASSO) regression. Predictive models were constructed using LR and multiple ML algorithms (Gaussian Naïve Bayes (GNB), Linear Discriminant Analysis (LDA), Random Forest (RF), eXtreme Gradient Boosting (XGBoost)). Model performance was evaluated using area under the curve (AUC), accuracy, sensitivity, specificity, and other metrics.

**Results:**

Hematoma clearance ratio was associated with 6- and 12-month outcomes. The Youden index suggested a threshold of 67.3%, whereas RCS analysis indicated an exploratory cut point near 81%; therefore, the 81% value should be considered hypothesis-generating. LR demonstrated internally validated performance comparable to the evaluated ML models in small-sample settings. Older age, higher ICH grade, and lower GCS were associated with a lower probability of favorable outcomes.

**Conclusion:**

In this single-center retrospective cohort, the hematoma clearance ratio was associated with long-term functional recovery after ICH surgery. LR provided performance comparable to the evaluated ML models and may serve as an interpretable research model.

## Introduction

Spontaneous intracerebral hemorrhage (sICH) is a devastating stroke subtype with high mortality and disability, causing substantial burden on healthcare systems and families worldwide (1). Despite advancements in surgical techniques and critical care management, a significant proportion of survivors are left with profound and permanent neurological deficits (2). The long-term functional outcome, often measured at 6 to 12 months post-ICH using scales such as the modified Rankin Scale (mRS), is a crucial endpoint for evaluating treatment efficacy and guiding rehabilitation strategies (3). Accurate long-term prognosis prediction is therefore essential for setting realistic expectations, informing clinical decisions, and identifying patients who will benefit most from aggressive surgery.

The pursuit of reliable prognostic tools has led to the development of various prediction models. Traditionally, prognostic models have relied on logistic regression (LR), valued for its simplicity and interpretable odds ratios (4, 5). More recently, machine learning (ML) algorithms—including Random Forest (RF), Support Vector Machines (SVM), and Neural Networks (NN)—have shown promise in capturing complex, non-linear relationships, often excelling with large, high-dimensional datasets (6). ML-based approaches have been increasingly applied across diverse clinical prediction domains, including COVID-19 severity prediction, genetic risk interpretation, cardiovascular disease characterization, and long-term survival prediction after hematopoietic cell transplantation (7–10), underscoring both the potential and the challenges of model interpretability and clinical translation.

Despite this progress, a critical review of the existing literature reveals several pronounced gaps. First, most studies focus on ICH incidence, in-hospital mortality, or specific complications (11–14), while long-term functional recovery remains underexplored — existing work at most covers 3-month outcomes (15, 16). Second, few studies have translated ML models into readily deployable and effective clinical tools (17). Third, high-performing ML models often function as black boxes, lacking the explainability needed for clinical trust and adoption (18). Crucially, many studies related with ICH operate with relatively small sample sizes, a constraint often overlooked in model development. While ML algorithms thrive on large datasets, their performance can be adversely affected by overfitting and increased variance in smaller cohorts, questioning their practical utility in such common research settings.

To address these gaps, this study aimed to develop and validate postoperative prognostic models for long-term functional outcome (mRS at 6 and 12 months) in surgically treated ICH patients. We conducted a head-to-head comparison between LR and multiple ML classifiers. We specifically tested the hypothesis that, with limited sample size, LR may achieve performance comparable to ML algorithms while providing superior interpretability for clinical application.

The manuscript is structured as follows: first, we describe the single-center surgical cohort, imaging-derived hematoma variables, outcome definitions, feature-selection strategy, and repeated internal validation framework; second, we compare LR with several commonly used ML classifiers; third, we interpret the final model using SHAP and restricted cubic spline (RCS) analyses; and finally, we discuss the clinical meaning and limitations of hematoma clearance ratio as a prognostic marker. Current knowledge remains limited by the scarcity of long-term post-surgical ICH prediction studies, the uncertain added value of complex ML models in small clinical cohorts, and the lack of interpretable and easily deployable prognostic tools. Our approach advances the field by directly comparing interpretable statistical modeling with ML algorithms under a modest-sample setting and by evaluating hematoma clearance ratio as a normalized surgical-efficiency metric. These findings improve the available knowledge by suggesting that a simple, interpretable LR model may achieve performance comparable to more complex ML models, while the exploratory clearance threshold identified in this study may help generate hypotheses for future prospective multicenter validation rather than define an established surgical target.

## Methods

### Study design and patient selection

This retrospective cohort study initially screened 1,021 consecutive patients diagnosed with sICH admitted to the Shengzhou Traditional Chinese Medicine Hospital between January 2021 and June 2024. From this initial screening, 234 patients met the preliminary inclusion criteria and were extracted from the hospital information system (HIS) for detailed evaluation.

The final study cohort of 185 patients was determined through rigorous application of predefined inclusion and exclusion criteria. Inclusion criteria comprised: (1) age ≥ 18 years; (2) complete clinical and follow-up data available; (3) sICH localized to basal ganglia or lobar regions. Exclusion criteria included: (1) presence of other severe neurological disorders or comorbidities including neurodegenerative diseases, intracranial space-occupying lesions, advanced heart failure; (2) secondary cerebral hemorrhage or subarachnoid hemorrhage; (3) prior surgical intervention before initial Computed Tomography (CT) imaging; (4) incomplete data or loss to follow-up.

**Figure 1:**
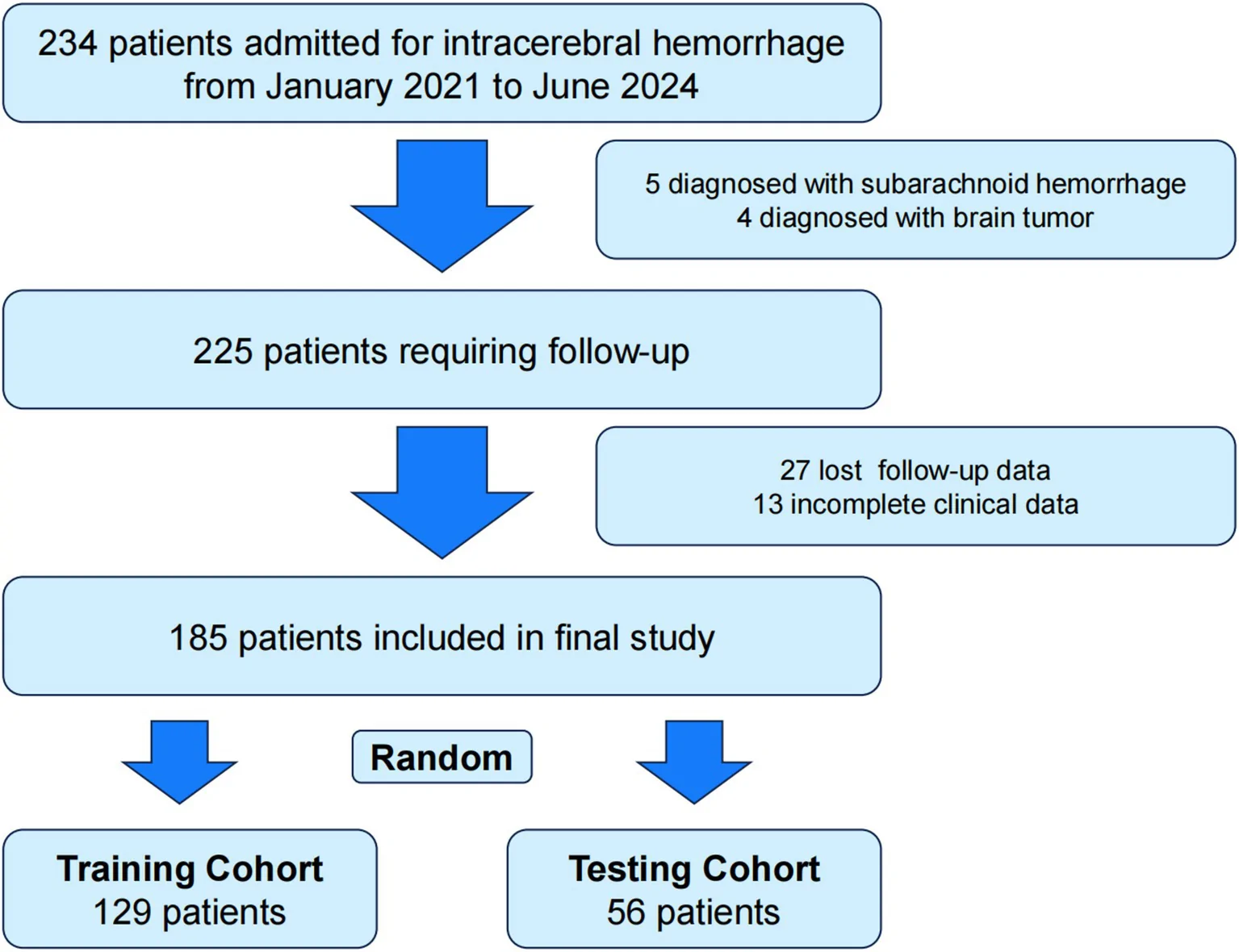
Selection flowchart of included and excluded patients. A flowchart detailing the inclusion of patients admitted for spontaneous intracerebral hemorrhage (sICH). After exclusions for specific diagnoses and missing data, 185 patients were included and randomly allocated into training and testing cohorts for the study.

A formal sample size calculation was performed during the study design phase. Based on the 21 candidate predictors considered for model development and anticipating approximately 50% rate of favorable outcomes, a minimum sample size of 210 patients was targeted to satisfy the general recommendation of 10 events per variable (EPV) (19). The final cohort of 185 patients, while slightly below this target, represents all eligible cases meeting our stringent criteria during the study period and is comparable to other single-center neurosurgical prediction studies. The flow chart of the selection process is presented in Figure 1. Then, the 185 patients included in the study were numbered in order of admission time and defined as the overall cohort dataset.

### Data acquisition

We collected characteristics such as general demographic characteristics, clinical characteristics, general imaging data of the patients and the procedure-related information. Demographic characteristics include age, gender and body mass index (BMI); Clinical characteristics include admission status, vascular risk factors such as smoking, alcohol; General imaging includes hematoma volume, hemorrhage location and so on; Procedure-related information include clearance ratio, surgical device and so on.

All patients were managed according to the American Heart Association spontaneous intracerebral hemorrhage guidelines (20). All patients underwent stereotactic surgery for hematoma evacuation. An mRS score of 0 to 3 was defined as a favorable outcome, indicating functional independence and significant surgical benefit (21).

### Assessment of clinical scores

Functional outcomes were assessed using the mRS at 3, 6, and 12 months after hospital discharge. To standardize the assessment of stroke severity and coma level, we employed established clinical scoring systems. The “ICH grade” corresponds to the conventional ICH Score, a published prognostic tool calculated as the sum of points assigned for age, Glasgow Coma Scale (GCS) score, ICH volume, presence of intraventricular hemorrhage (IVH), and infratentorial origin of hemorrhage (22). The GCS was used to evaluate the level of consciousness, and its scoring was performed according to standard criteria, assessing the best eye, verbal, and motor responses (23). The details of these scoring systems are provided in the Supplementary Tables 1–3.

Favorable functional outcome was defined as mRS 0–3, and unfavorable outcome was defined as mRS 4–6. For all multivariable models, the binary outcome was coded as 1 for favorable outcome and 0 for unfavorable outcome.

### Imaging analysis and volumetric assessment

The hematoma volume was quantitatively measured using 3D Slicer software on pre-operative and post-operative non-contrast computed tomography (NCCT) scans. The hematoma clearance ratio was subsequently calculated as follows:

$\begin{array}{l} \text{Clearance Ratio}=(\begin{array}{l} \text{Preoperative Volume}-\\ \text{Postoperative Volume} \end{array})/\\ \text{Preoperative Volume}. \end{array}$

### Feature selection and model construction

To reduce the risk of information leakage, all preprocessing, feature selection, model tuning, and model evaluation procedures were performed within a repeated internal validation framework (Supplementary Figure 1). In each iteration, the full cohort was randomly divided into a training set and a held-out validation set at a 70:30 ratio using stratified sampling according to outcome status. Missing-value imputation was fitted within the training set only and then applied to the corresponding validation set. Continuous predictors were standardized using z-score normalization, with the mean and standard deviation estimated from the training set only and then applied to the validation set. Model performance was reported as the mean of all 100 iterations, ensuring reliable estimation of generalization ability while mitigating overfitting concerns inherent in modest sample sizes.

Feature selection was performed within the training set using Least Absolute Shrinkage and Selection Operator (LASSO) regression with the one-standard-error rule. Hyperparameters for the evaluated models were optimized using 5-fold cross-validation within the training set. The final model in each iteration was fitted on the training set and evaluated once on the corresponding held-out validation set. This procedure was repeated 100 times, and model performance was reported as the mean value across the 100 held-out validation sets.

Predictive models were constructed using logistic regression (LR), Gaussian Naïve Bayes (GNB), Linear Discriminant Analysis (LDA), Random Forest (RF), and eXtreme Gradient Boosting (XGBoost). The primary performance estimate was defined as the mean AUC on the held-out validation sets across the 100 repeated 70:30 splits.

### Model selection and model interpretation

Model performance was assessed by the area under the curve (AUC) of the subject operating characteristics (ROC), accuracy, sensitivity, specificity, positive predictive value (PPV), negative predictive value (NPV), F1 score and the Youden index. Favorable functional outcome was treated as the positive class for all classification metrics.

The SHapley Additive exPlanation (SHAP) approach was applied to illustrate the contribution of individual variables, clarifying their influence on the predictive model (24). For key continuous predictors identified through SHAP analysis, we further conducted restricted cubic spline (RCS) analysis to characterize their potential non-linear relationships with favorable functional outcomes. The ROC-derived threshold was determined using the Youden index, which maximizes the sum of sensitivity and specificity. Separately, the RCS-derived candidate cut point was estimated from the fitted spline curve as the point of maximal curvature, defined as the point with the largest absolute second derivative of the fitted log-odds curve across the observed range of hematoma clearance ratio.

### Calibration and decision-curve analysis

Calibration of the final LR model was assessed using calibration plots and Brier scores. Calibration plots compared predicted probabilities with observed outcome rates across grouped predicted-risk intervals, with the dashed diagonal line representing perfect calibration. The Brier score was calculated as the mean squared difference between predicted probability and observed outcome status, with lower values indicating better overall prediction accuracy.

Clinical utility was evaluated using decision-curve analysis. Because the LR model predicted the probability of favorable functional outcome, decision-curve analysis was performed after converting the predicted probability into the estimated risk of unfavorable outcome. Net benefit was calculated across threshold probabilities and compared with treat-all and treat-none strategies.

### Web calculator development and internal validation

After the final LR model was established, an online web calculator was developed using R Shiny. The calculator was based on the final 12-month LR model and included four input variables: age, admission GCS score, ICH grade, and hematoma clearance ratio. Age was entered in years, GCS and ICH grade were entered as original clinical scores, and hematoma clearance ratio was entered as a proportion ranging from 0 to 1 rather than as a percentage from 0 to 100.

The backend of the calculator used the final LR equation to calculate the log-odds of favorable functional outcome and converted it into predicted probability. The web calculator was developed after model specification was finalized and did not influence feature selection, model fitting, or model evaluation.

To verify technical consistency, we performed representative test-case checking to confirm that the web calculator output was consistent with the underlying LR equation. Because no external or prospective validation cohort was available, the calculator should be regarded as an internally validated research tool rather than a clinically established decision-support system.

### Statistical analysis

Statistical evaluations and model construction were conducted with R software (version 4.5.1). Missing data in predictors were limited (range: 0–3.2%; involving IVH and spot sign). Within each resampling iteration, missing-value imputation was fitted using the training set only and then applied to the corresponding held-out validation set. Outcome variables had no missing data and were not imputed. Normally distributed continuous variables were represented as means and standard deviations (SD), and medians (m) and inter-quartile range for non-normally distributed data. Categorical variables were presented as numbers (no.) and percentages (%). Differences between continuous variables were compared using t-tests or Mann–Whitney U test, and differences in categorical variables using Chi-square tests. A *p*-value under 0.05 (two-tailed) was considered indicative of statistical significance.

## Results

### Baseline characteristics and outcomes

In this study, we analyzed data from 185 patients with sICH (Table 1). At the 6-month follow-up, 98 (53.0%) patients showed a favorable outcome, increasing to 112 (60.5%) at 12 months. Notably, patients with favorable outcomes at 6 months were significantly younger (54 ± 11 years) compared to those with unfavorable outcomes (61 ± 15 years, *p* < 0.001). Additionally, patients with favorable outcomes had higher admission GCS scores (10.9 ± 3.1) compared to those with unfavorable outcomes (10.0 ± 3.1, *p* = 0.008). The ICH grade was also significantly different, with fewer patients in the highest grade (4–5) in the favorable outcome group (0%) compared to the unfavorable group (6.4%, *p* = 0.003). This difference became more pronounced at the 12-month follow-up, with the proportion of highest ICH grade remaining at 0% in the favorable group but increasing to 16.4% in the unfavorable outcome group. Moreover, the clearance ratio was significantly higher in patients with favorable outcomes at both 6 months (77%, *p* < 0.001) and 12 months (78%, *p* < 0.001).

**Table 1:** Demographics and clinical characteristics of study.

Characteristic	Overall *N* = 185	6-months outcome			12-months outcome		
		No *N* = 87	Yes *N* = 98	*p*-value^1^	No *N* = 73	Yes *N* = 112	*p*-value^1^
Demographics							
Gender				0.14			0.087
Male	138 (75%)	60 (69%)	78 (80%)		49 (67%)	89 (79%)	
Female	47 (25%)	27 (31%)	20 (20%)		24 (33%)	23 (21%)	
Age (years)	58 ± 14	61 ± 15	54 ± 11	**<0.001**	63 ± 16	54 ± 11	**<0.001**
BMI (kg/cm^2^)	24.0 (21.5, 26.7)	24.2 (21.7, 26.5)	23.9 (21.4, 27.3)	>0.9	23.9 (21.5, 26.2)	24.1 (21.6, 27.5)	0.3
Admission status							
GCS	11.0 (8.0, 13.0)	10.0 (7.0, 13.0)	11.0 (9.0, 14.0)	0.074	10.0 (7.0, 12.0)	11.0 (9.0, 14.0)	**0.008**
ICH grade				**0.003**			**<0.001**
0	10 (5.4%)	3 (3.4%)	7 (7.1%)		3 (4.1%)	7 (6.3%)	
1	48 (26%)	17 (20%)	31 (32%)		13 (18%)	35 (31%)	
2	77 (42%)	35 (40%)	42 (43%)		28 (38%)	49 (44%)	
3	38 (21%)	20 (23%)	18 (18%)		17 (23%)	21 (19%)	
4	11 (5.9%)	11 (13%)	0 (0%)		11 (15%)	0 (0%)	
5	1 (0.5%)	1 (1.1%)	0 (0%)		1 (1.4%)	0 (0%)	
Vascular risk factors							
Alcohol use	74 (40%)	35 (40%)	39 (40%)	>0.9	28 (38%)	46 (41%)	0.8
Smoke (>3 years)	79 (43%)	30 (34%)	49 (50%)	**0.048**	25 (34%)	54 (48%)	0.084
Hypertension	117 (63%)	58 (67%)	59 (60%)	0.4	48 (66%)	69 (62%)	0.7
Diabetes	16 (8.6%)	11 (13%)	5 (5.1%)	0.12	10 (14%)	6 (5.4%)	0.088
Anticoagulant use				**0.013**			0.067
None	166 (90%)	72 (83%)	94 (96%)		61 (84%)	105 (94%)	
Antiplatelet	10 (5.4%)	8 (9.2%)	2 (2.0%)		7 (9.6%)	3 (2.7%)	
Anticoagulant	9 (4.9%)	7 (8.0%)	2 (2.0%)		5 (6.8%)	4 (3.6%)	
Hemorrhage characteristics							
Hemorrhage location				0.5			0.3
Deep	157 (85%)	75 (86%)	82 (84%)		62 (85%)	95 (85%)	
Lobar	15 (8.1%)	5 (5.7%)	10 (10%)		4 (5.5%)	11 (9.8%)	
Deep + Lobar	13 (7.0%)	7 (8.0%)	6 (6.1%)		7 (9.6%)	6 (5.4%)	
Preoperative hematoma volume(mL)	42 (30, 59)	44 (31, 62)	39 (29, 57)	0.090	43 (31, 65)	41 (30, 56)	0.2
Admission SBP (mmHg)	157 (138, 179)	156 (137, 180)	159 (140, 179)	0.9	156 (135, 180)	158 (140, 179)	>0.9
Midline shift	152 (82%)	73 (84%)	79 (81%)	0.7	62 (85%)	90 (80%)	0.5
Spot Sign	7 (3.8%)	4 (4.6%)	3 (3.1%)	0.9	4 (5.5%)	3 (2.7%)	0.6
IVH	79 (43%)	46 (53%)	33 (34%)	**0.015**	38 (52%)	41 (37%)	0.061
Procedure-related information							
Surgical device				0.2			**0.033**
Frame	46 (25%)	18 (21%)	28 (29%)		12 (16%)	34 (30%)	
Brainlab	103 (56%)	55 (63%)	48 (49%)		49 (67%)	54 (48%)	
Remebot	36 (19%)	14 (16%)	22 (22%)		12 (16%)	24 (21%)	
Onset-to-surgery time(h)	50 (30, 82)	47 (30, 84)	50 (29, 82)	0.8	51 (32, 84)	49 (29, 82)	0.8
Procedure duration(min)	51 (45, 60)	55 (45, 63)	50 (40, 60)	0.070	55 (45, 61)	51 (44, 60)	0.3
Postoperative hematoma volume(mL)	11 (5, 22)	14 (7, 27)]	9 (4, 15)	**<0.001**	16 (9, 28)	9 (5, 15)	**<0.001**
Clearance ratio	0.73 (0.56, 0.85)	0.63 (0.50, 0.81)	0.77 (0.64, 0.86)	**<0.001**	0.60 (0.50, 0.76)	0.78 (0.64, 0.86)	**<0.001**

Bold values indicate statistical significance.

^1^T test (Age); Mann–Whitney U test (Other continuous); Chi-square test (All categorical). BMI, Body Mass Index; GCS, Glasgow Coma Scale; ICH, Intracerebral Hemorrhage; IVH, Intraventricular Hemorrhage; SBP, Systolic Blood Pressure.

These findings suggest that younger age, higher GCS scores, lower ICH grades and higher clearance ratio are associated with better outcomes at both 6- and 12-months post-ICH.

### Univariate predictive performance of candidate variables

**Figure 2:**
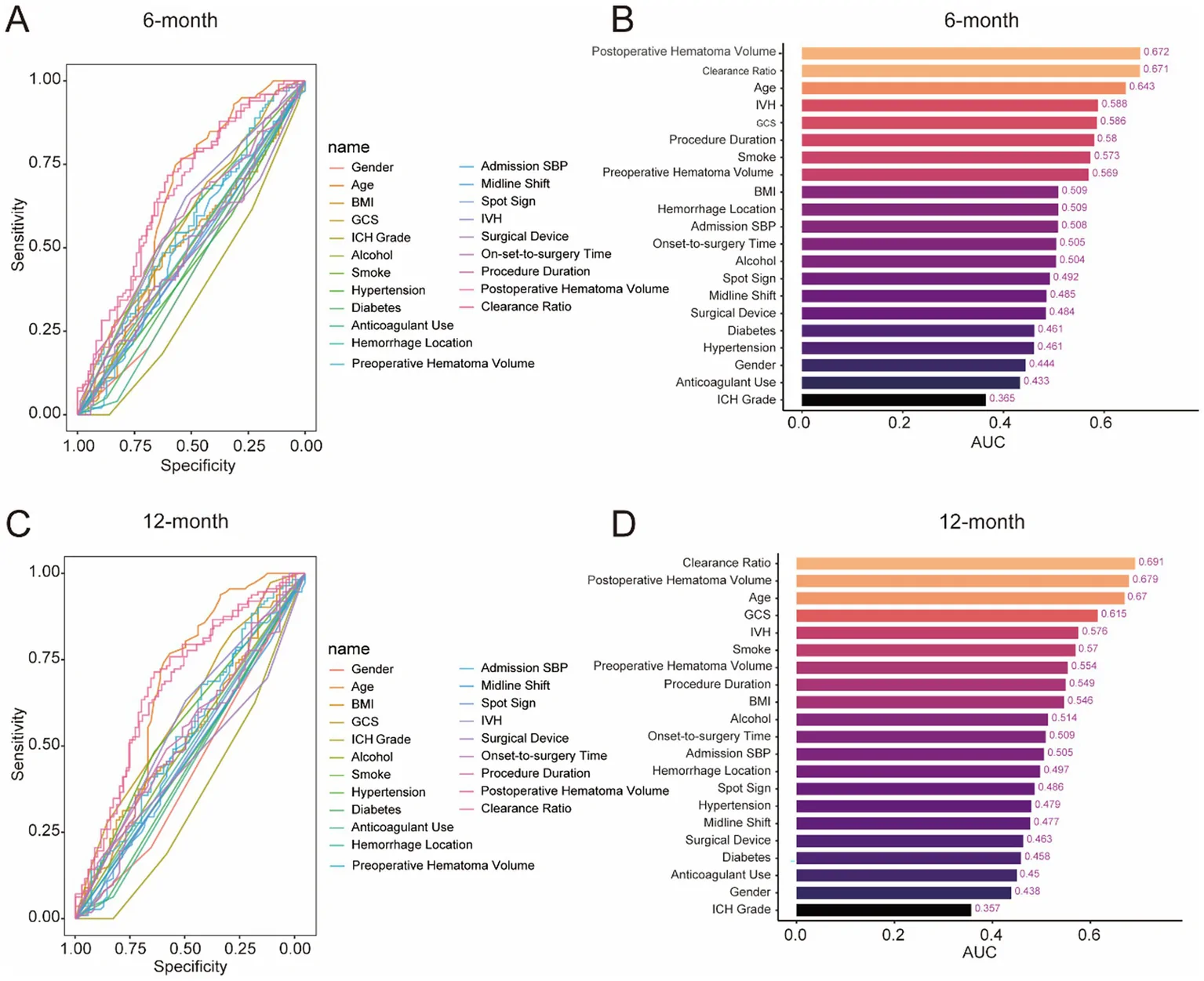
Univariate analysis of variables for predicting 6-month and 12-month favorable outcomes. **(A,C)** Receiver operating characteristic (ROC) curves for each variable; **(B,D)** Area under curve (AUC) bar graphs demonstrating the discriminative ability of each variable. GCS, Glasgow Coma Scale; ICH, Intracerebral Hemorrhage; IVH, Intraventricular Hemorrhage; SBP, Systolic Blood Pressure.

We first evaluated the individual predictive power of each variable for both 6-month and 12-month favorable functional outcome (Figure 2). The analysis revealed that the top-performing predictors were consistent across both time points: Postoperative hematoma volume (6-month AUC: 0.672; 12-month AUC: 0.679), age (6-month AUC: 0.643; 12-month AUC: 0.670) and clearance ratio (6-month AUC: 0.671; 12-month AUC: 0.691) demonstrated the strongest predictive utility. The clearance ratio showed a slightly higher predictive ability for the 12-month outcome (AUC: 0.691) than for the 6-month outcome (AUC: 0.671). Overall, the vast majority of variables demonstrated moderate or limited predictive power (AUC < 0.6) at both time points, yet their relative rankings remained largely consistent.

### Assessment of multicollinearity

**Figure 3:**
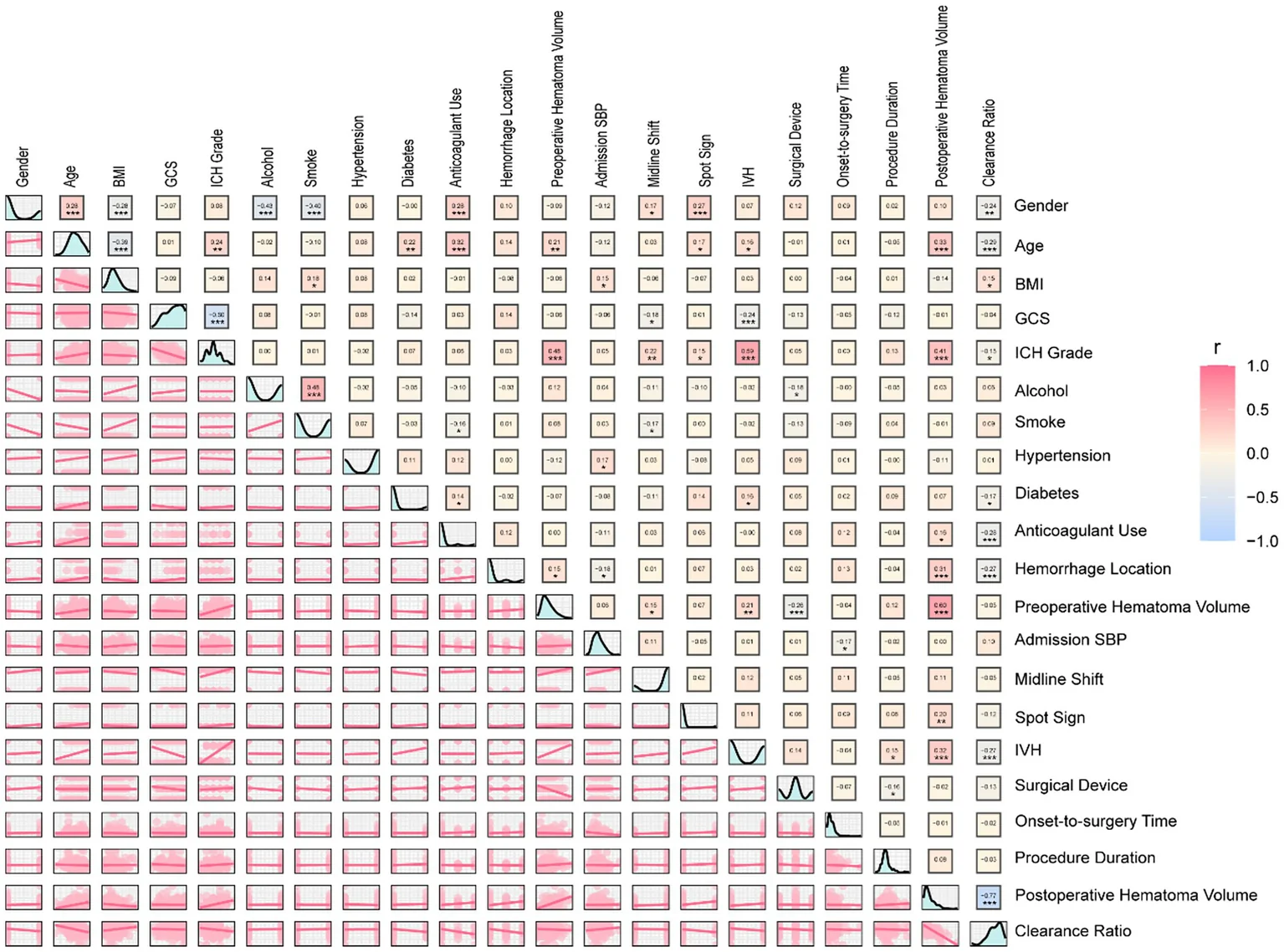
Correlation matrix of candidate variables. The matrix integrates a heatmap and scatter plots, with correlations calculated appropriate to variable types.

Prior to variable selection with Lasso regression, an analysis was conducted to assess multicollinearity among all 21 candidate variables. Corresponding correlation coefficients were employed based on variable types: Spearman’s correlation for continuous-continuous pairs, Cramer’s V for categorical-categorical pairs, and point-biserial correlation for continuous-categorical pairs. The results demonstrated that the absolute values of all correlation coefficients were below the critical threshold of 0.8 (Figure 3). This finding indicates only weak linear associations and an absence of significant multicollinearity among the candidate variables. Therefore, all 21 variables were deemed suitable for subsequent screening via Lasso regression.

### Feature selection

The patients were randomly divided into the training set and the validation set in a 7:3 ratio. The training set was used to establish the model, and the validation set was used to test the model established. There were no statistically significant differences in all variables included between the two groups (*p* > 0.05), which showed that the data grouping was random and reasonable (Supplementary Table 4).

**Figure 4:**
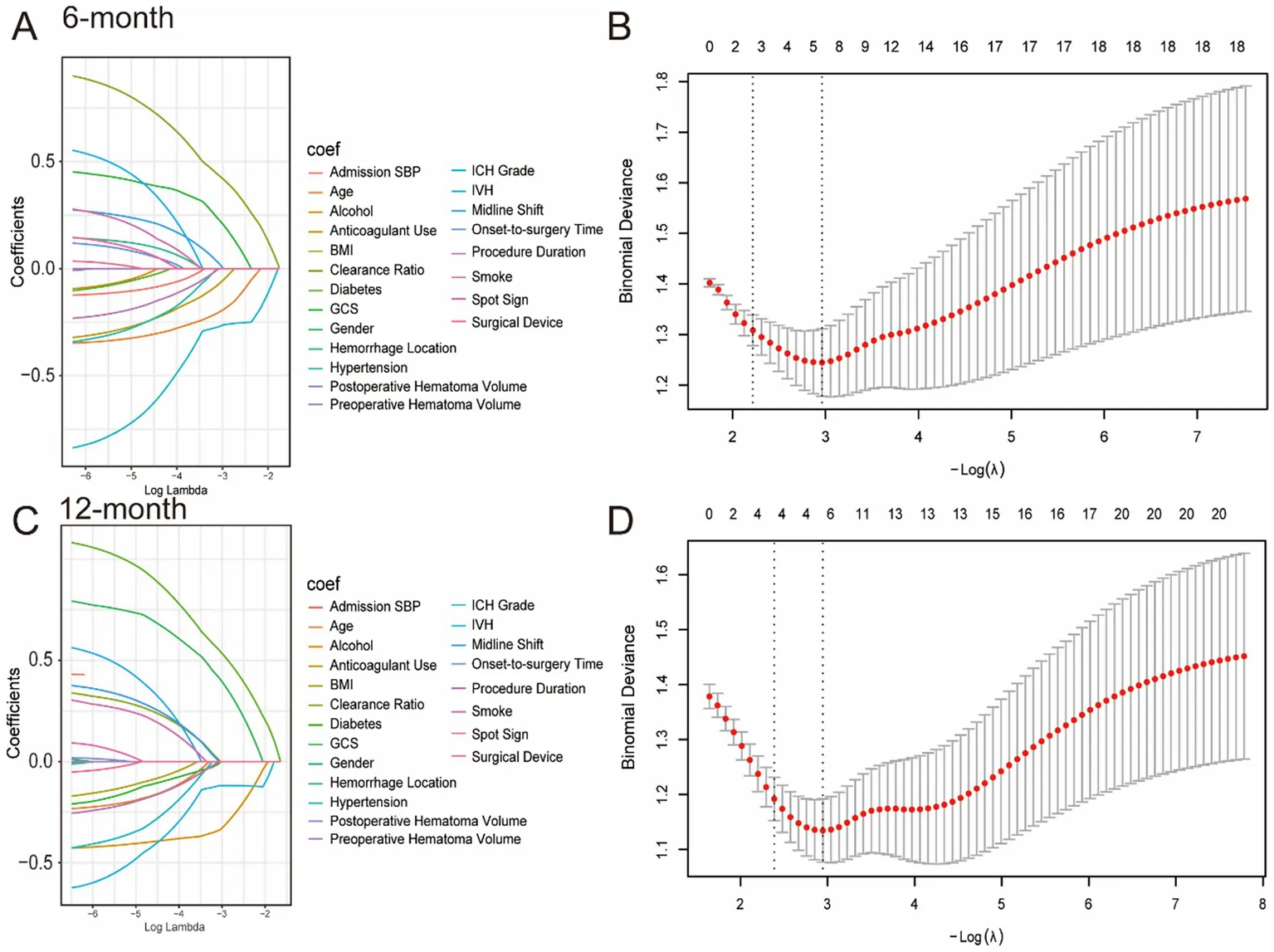
Variable selection via Least Absolute Shrinkage and Selection Operator (Lasso) regression with the one-standard-error rule. **(A,C)** Coefficient paths showing variable shrinkage against *λ*; **(B,D)** Cross-validation error curve identifying the optimal *λ* (*λ*.1se) for selecting the core predictor sets for 6-month and 12-month outcomes.

Variables selected by Lasso regression with the one-standard-error rule for 6-month and 12-month prognosis are presented in Figure 4. The optimal models were achieved at *λ* = 0.106 (log(λ) = −2.24) for 6-month prognosis and λ = 0.091 (log(λ) = −2.40) for 12-month prognosis. The screened variables included age, ICH grade and clearance ratio in 6-month model, while in 12-month model they included age, ICH grade, clearance ratio and GCS.

The GCS at admission has long been deemed a strong predictor of functional outcomes and mortality in patients with ICH. So, it was also included in the final model. Based on the variable selection results and clinical relevance, four key predictors—age, ICH grade, clearance ratio, and GCS—were ultimately identified as the consensus feature set, whereas variables such as Postoperative hematoma volume, despite showing moderate univariate association, were not selected by the Lasso algorithm for the multivariate model. These variables were subsequently used to construct and compare LR and ML-based prognostic models.

### Multiple machine learning model performance

**Figure 5:**
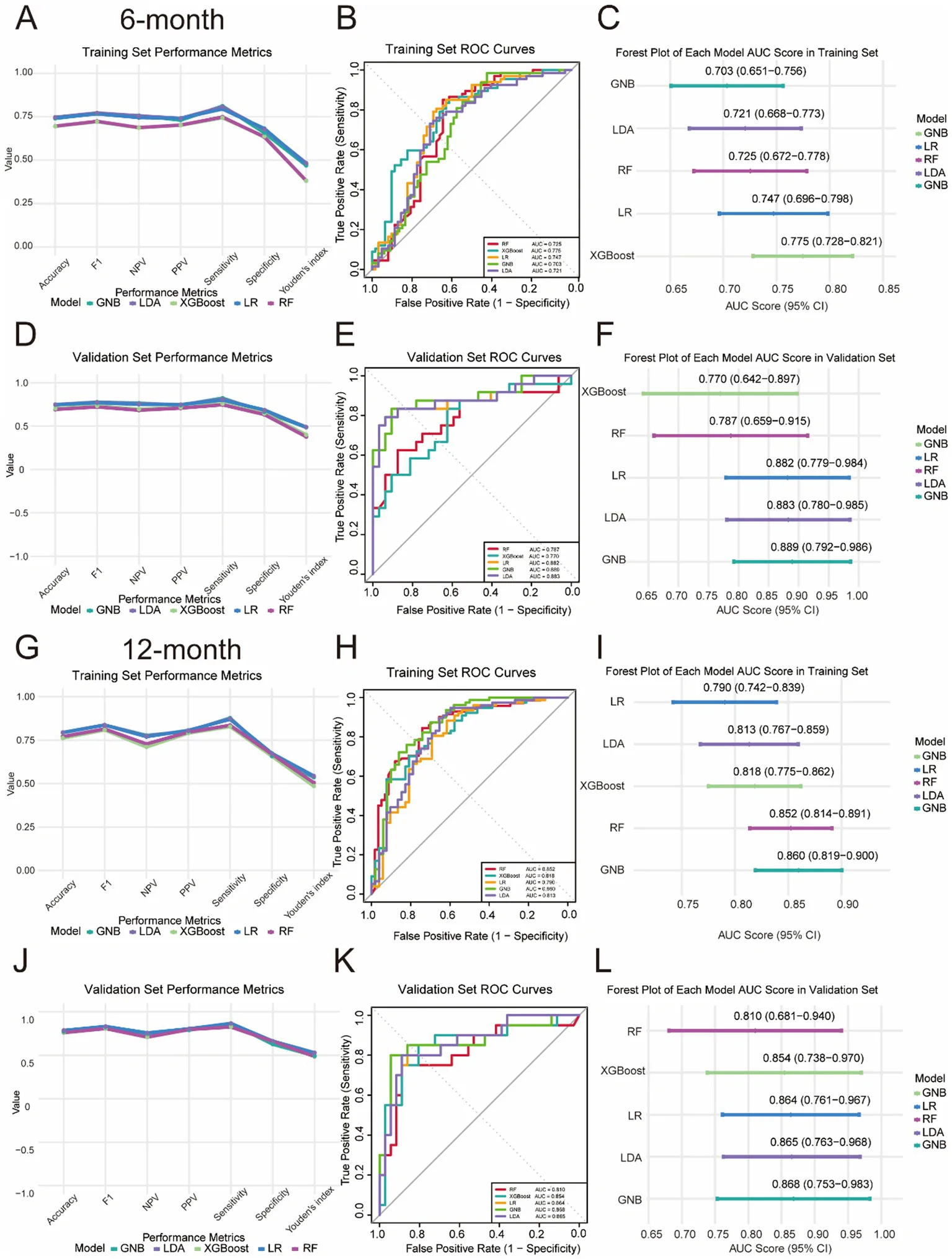
Performance evaluation of multiple machine learning models for predicting 6-month and 12-month favorable outcomes. **(A–C)** Performance on the training set for 6-month outcome prediction: **(A)** Line plots of performance metrics across hyperparameter tuning; **(B)** Receiver operating characteristic (ROC) curves; **(C)** Forest plot of the area under the curve (AUC) values with 95% confidence intervals. **(D–F)** Performance on the test set for 6-month outcome prediction, with the same subplot descriptions as **(A–C)**. **(G–I)** Performance on the training set for 12-month outcome prediction. **(J–L)** Performance on the test set for 12-month outcome prediction. RF, Random Forest; XGBoost, extreme Gradient Boosting; LR, Logistic Regression; GNB, Gaussian Naïve Bayes; LDA, Linear Discriminant Analysis; PPV, Positive Predictive Value; NPV, Negative Predictive Value; F1, F1-score.

Multiple models were evaluated for predicting 6-month and 12-month favorable functional outcomes. The primary performance estimate was the mean performance on held-out validation sets across 100 repeated 70:30 splits, as shown in Table 2 and Figure 5.

**Table 2:** Performance metrics of 6-month and 12-month models.

Models	Sensitivity	Specificity	Accuracy	PPV	NPV	F1	Youden index
6-month model							
RF	0.74	0.63	0.69	0.71	0.68	0.72	0.38
XGBoost	0.76	0.64	0.70	0.71	0.70	0.73	0.40
LR	0.80	0.69	0.74	0.75	0.75	0.77	0.48
GNB	0.82	0.66	0.75	0.74	0.76	0.78	0.48
LDA	0.81	0.68	0.75	0.75	0.76	0.77	0.49
12-month model							
RF	0.82	0.66	0.76	0.80	0.71	0.81	0.48
XGBoost	0.83	0.66	0.76	0.80	0.71	0.81	0.49
LR	0.87	0.66	0.79	0.81	0.76	0.83	0.53
GNB	0.86	0.63	0.77	0.79	0.75	0.82	0.49
LDA	0.86	0.66	0.79	0.81	0.76	0.83	0.53

RF, Random Forest; XGBoost, extreme Gradient Boosting; LR, Logistic Regression; GNB, Gaussian Naïve Bayes; LDA, Linear Discriminant Analysis; PPV, Positive Predictive Value; NPV, Negative Predictive Value; F1, F1-score. TP, True Positive; TN, True Negative; FP, False Positive; FN, False Negative. Sensitivity = TP/(TP + FN); Specificity = TN/(TN + FP); Accuracy = (TP + TN)/(TP + TN + FP + FN); PPV = TP/(TP + FP); NPV = TN/(TN + FN); Youden index = Sensitivity+Specificity-1.

For 6-month favorable outcome prediction, the evaluated models demonstrated broadly comparable performance in repeated internal validation. LR achieved a mean validation AUC of 0.75, with a sensitivity of 0.799, specificity of 0.685, and Youden index of 0.484. It was closely followed by LDA (Youden index: 0.489) and GNB (Youden index: 0.485, AUC: 0.70), with all three models showing nearly identical discriminative ability. Ensemble methods yielded more modest results in this limited-sample cohort.

For 12-month favorable outcome prediction, LR achieved a mean validation AUC of 0.79, with a sensitivity of 0.868 and Youden index of 0.529. LDA showed comparable performance, whereas GNB demonstrated slightly lower overall performance.

LR was selected as the final interpretable postoperative prognostic model, not because it clearly outperformed LDA, but because it achieved comparable internally validated performance while offering greater clinical interpretability. Unlike LDA, whose coefficients primarily define a discriminant score for class separation, LR directly models the log-odds and predicted probability of favorable functional outcome. Therefore, LR coefficients can be transformed into odds ratios with 95% confidence intervals, allowing clinicians to understand the direction and magnitude of each predictor’s association with outcome. In addition, the LR equation can be transparently implemented in a web calculator and used to generate individualized predicted probabilities.

### Model interpretation and variable importance

**Figure 6:**
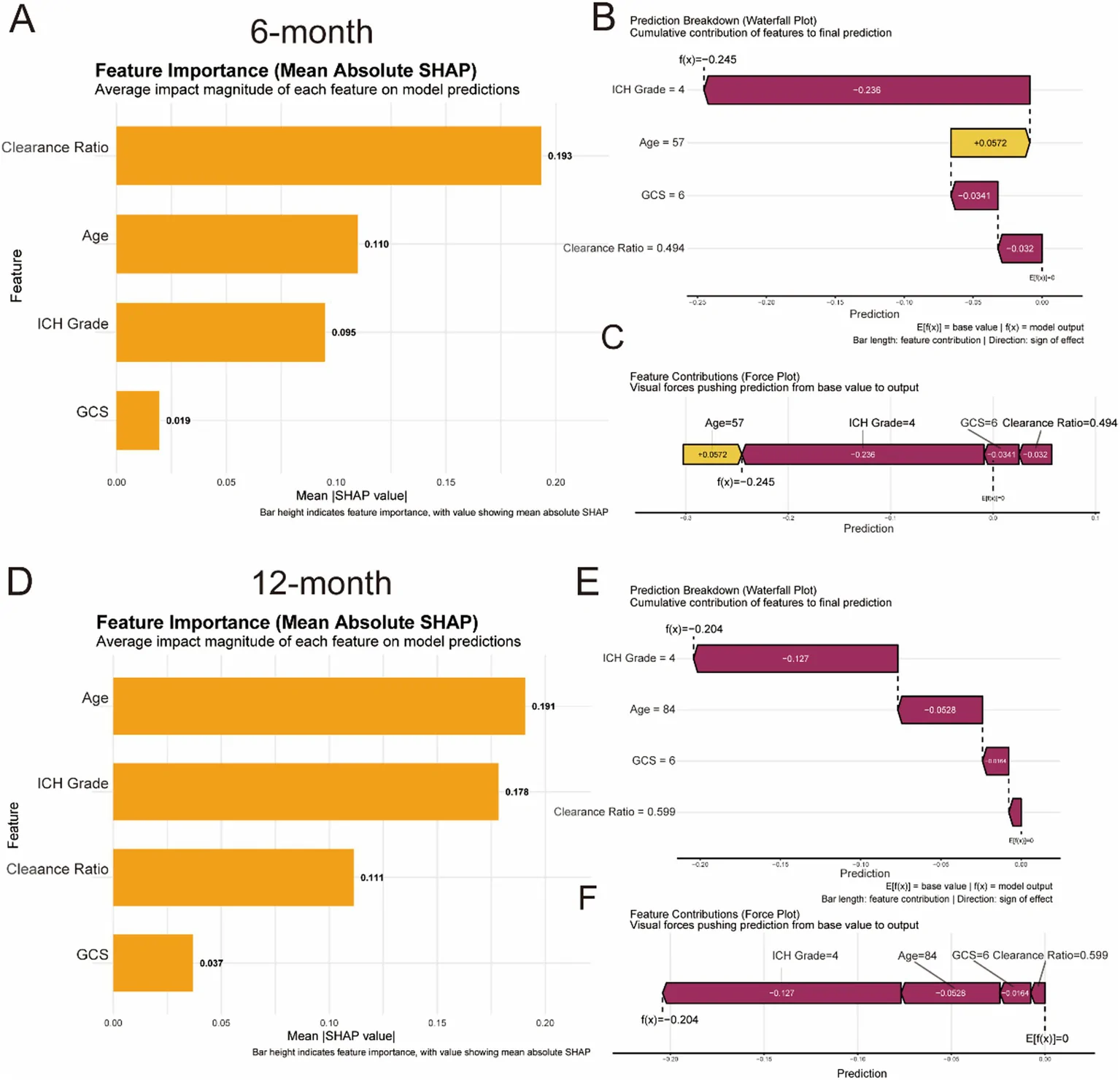
SHAP explainability plots for the 6-month and 12-month Logistic Regression (LR) models. **(A,D)** Summary plots of feature importance. **(B,E)** Waterfall plots illustrating the additive contribution of each feature to the model’s log-odds output for a single prediction. *f(x)* denotes the final log-odds of a good outcome. **(C,F)** Force plots visualizing the same prediction as in **(B,E)**. Features in yellow (pushing right) increase the log-odds of a favorable outcome, while features in purple (pushing left) decrease it. SHAP, SHapley Additive exPlanation.

The SHAP summary plots (Figures 6A,D) illustrate the relative importance of features in the LR models identifying several consistent and time-specific predictors. Subsequent RCS analyses were performed on the selected continuous variables. For the 12-month model, age (mean |SHAP| = 0.191), ICH Grade (0.178), and clearance ratio (0.111) emerged as the most influential features. Notably, the 6-month model highlighted a stronger contribution from the hematoma clearance ratio (0.193), aligning with the nonlinear relationship and exploratory RCS-derived candidate reference point.

To elucidate the individual contribution of these variables, we generated SHAP waterfall plots (Figures 6B,E) and force plots (Figures 6C,F) for representative patients. In these plots, the model output f(x) is shown on the log-odds scale for a favorable outcome. The waterfall plot for the 6-month model demonstrates how a higher ICH grade and a lower GCS score—both yielding negative SHA*p* values—collectively push the model’s prediction away from a favorable outcome, resulting in a final output of f(x) = −0.25 log-odds. A similar interpretation applies to the 12-month explanation.

The force plots provide a complementary view: features pushing the prediction to the right (in yellow) increase the probability of a favorable outcome, whereas those pushing to the left (in purple) decrease it.

**Figure 7:**
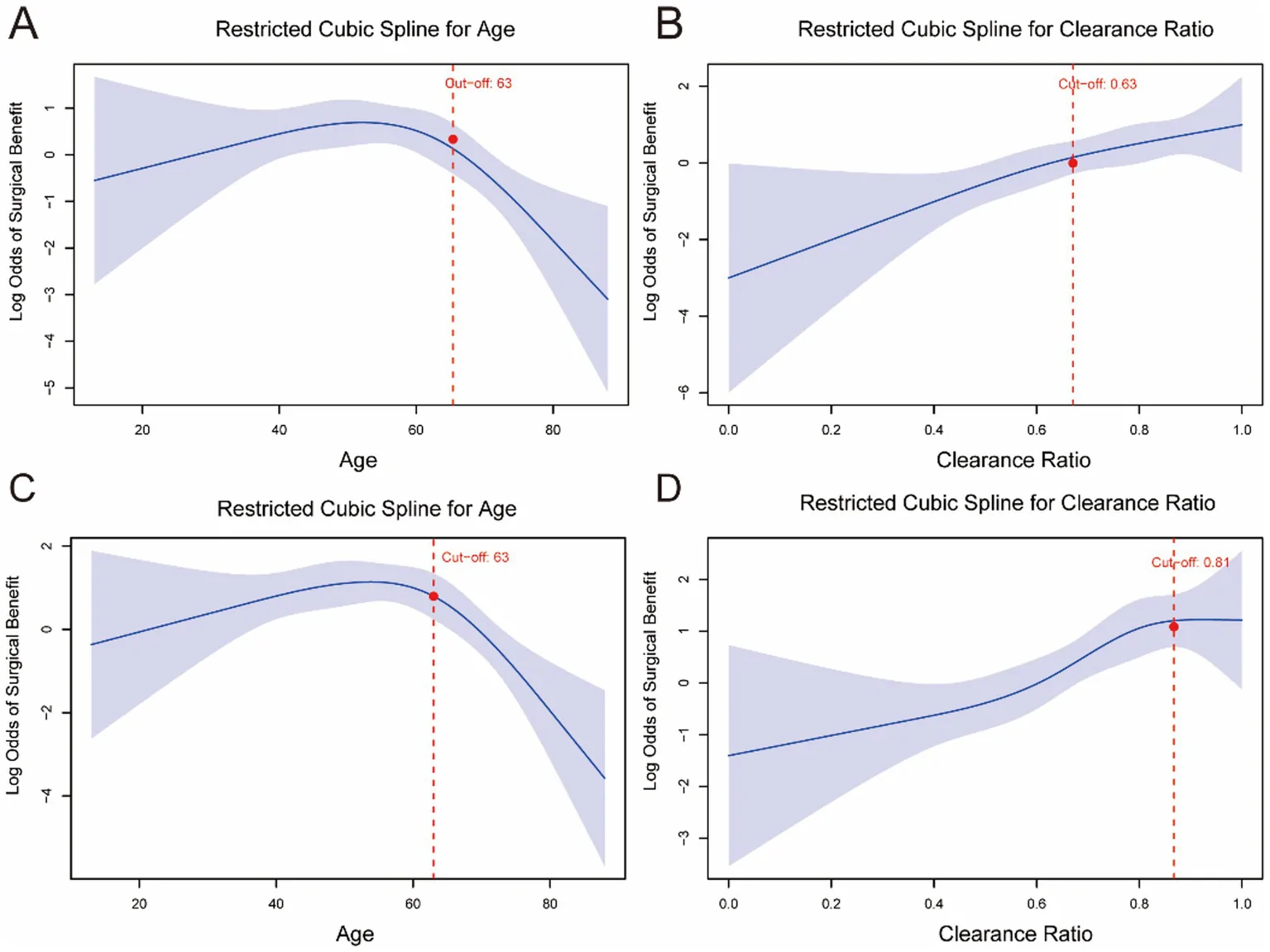
Nonlinear effects of key predictors. **(A,B)** Restricted cubic spline (RCS) curves for age and clearance ratio in the 6-month model. **(C,D)** Corresponding analyses for the 12-month model. Purple shaded areas indicate 95% confidence intervals; Red vertical lines denote clinically relevant thresholds determined by inflection point analysis of the RCS curves.

These visualizations, contextualized with RCS-derived thresholds (Figure 7), enhance clinical interpretability of both time-specific models, offering actionable insights into critical factors influencing recovery at 6 and 12 months. For hematoma clearance ratio, two exploratory reference values were identified using different statistical criteria. The ROC-Youden method yielded a threshold of 67.3%, which maximized the combined sensitivity and specificity. In contrast, the RCS maximal-curvature analysis identified an approximate candidate reference point near 81.0%, corresponding to the point at which the fitted log-odds curve showed the greatest local change.

**Figure 8:**
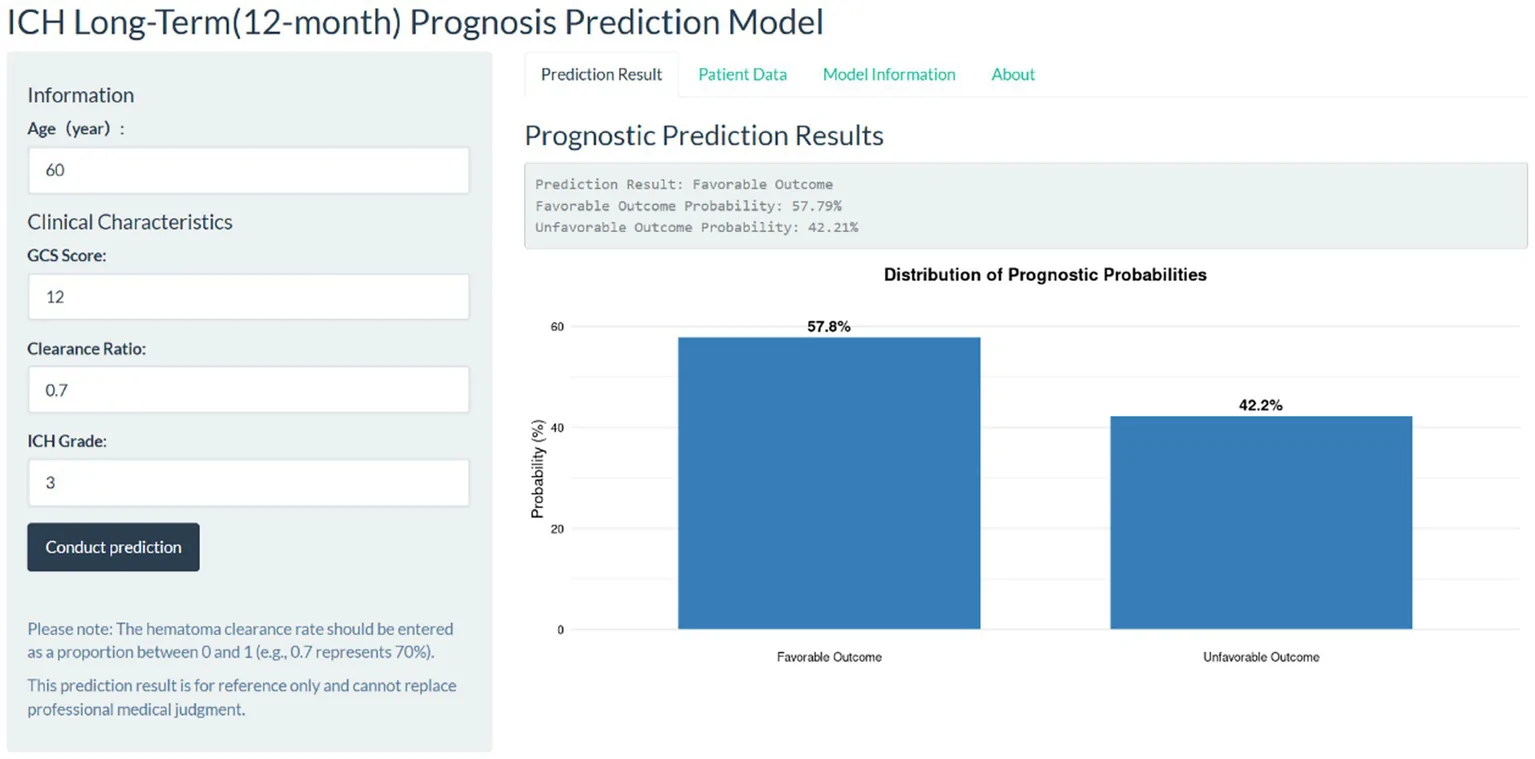
A web-based calculator for predicting long-term prognosis in patients with spontaneous intracerebral hemorrhage (sICH). The web calculator deployed on 1st, September 2025 was constructed based on the final logistic regression model for 12-month outcome prediction.

Furthermore, a web calculator was constructed based on the selected 4 indicators, providing postoperative estimation of favorable long-term outcome after hematoma evacuation^1^ (Figure 8). The web calculator was constructed based on the final LR model for 12-month outcome prediction. It is specified as:

$\begin{array}{l} \text{logit}(p)=-2.85825+5.89391^{*}\text{Clearance Ratio}-0.23346^{*}\\ \mathrm{ICH}\;\text{Grade}+0.16986^{*}\mathrm{GCS}-0.03235^{*}\mathrm{Age}. \end{array}$

The full specification of the final 12-month LR model is presented in Table 3, including the intercept, regression coefficients, odds ratios, 95% confidence intervals, *p* values, and predictor scales. The model was fitted using the original clinical scales for the final clinical equation.

**Table 3:** Full specification of the final 12-month logistic regression model.

Predictor	β coefficient	Odds ratio (OR)	95% CI	*p* value	Clinical interpretation
Intercept	−2.85825	—	—	—	Baseline log-odds when all predictors are zero
Hematoma clearance ratio	5.89391	362.82	[35.9,3666.5]	<0.001	Higher clearance ratio was associated with a higher probability of 12-month favorable functional outcome
ICH grade	−0.23346	0.79	[0.50,1.26]	0.324	Higher ICH grade was associated with a lower probability of favorable outcome
Admission GCS score	0.16986	1.19	[1.03,1.36]	0.017	Higher GCS score was associated with a higher probability of favorable outcome
Age	−0.03235	0.97	[0.94,0.99]	0.023	Older age was associated with a lower probability of favorable outcome

LR, logistic regression; OR, odds ratio; CI, confidence interval; GCS, Glasgow Coma Scale; ICH, intracerebral hemorrhage; mRS, modified Rankin Scale.

### Calibration and clinical utility of the final LR model

**Figure 9:**
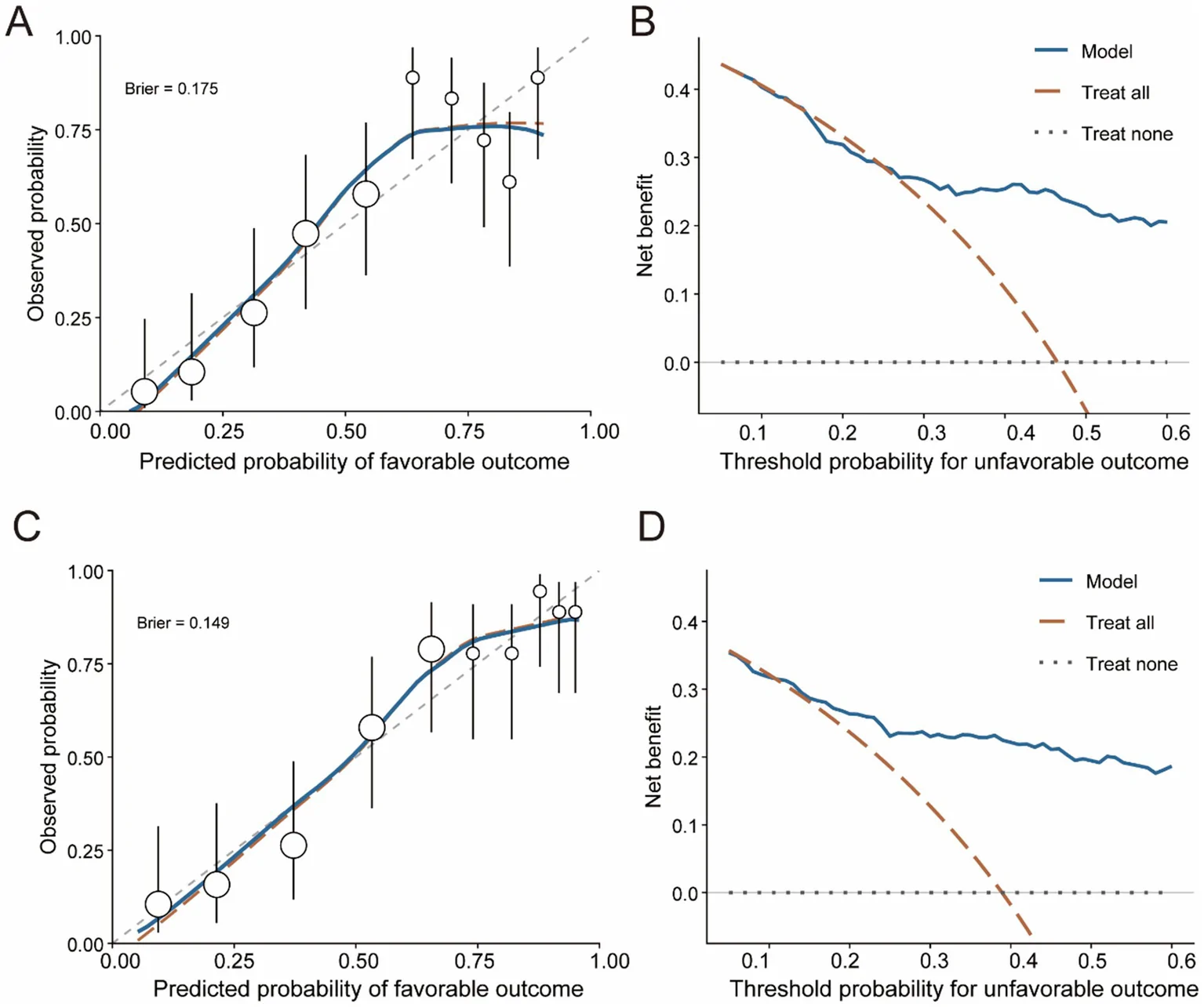
Calibration and decision-curve analysis of the final LR model. **(A)** Calibration plot for 6-month favorable functional outcome prediction. **(B)** Decision-curve analysis for 6-month unfavorable outcome risk. **(C)** Calibration plot for 12-month favorable functional outcome prediction. **(D)** Decision-curve analysis for 12-month unfavorable outcome risk. In calibration plots, the dashed diagonal line indicates perfect calibration, and circles indicate grouped observed outcome rates with confidence intervals. Decision-curve analysis compares the net benefit of the LR model with treat-all and treat-none strategies across threshold probabilities.

Calibration and decision-curve analyses were performed for the final LR models (Figure 9). The 6-month model showed a Brier score of 0.175, and the 12-month model showed a Brier score of 0.149. Calibration plots demonstrated the relationship between predicted and observed probabilities of favorable functional outcome. Decision-curve analysis suggested that the LR model provided greater net benefit than the treat-none strategy across the evaluated threshold range and showed potential clinical utility for postoperative risk stratification. Because these analyses were performed in a single-center retrospective cohort without external validation, they should be interpreted as internal exploratory assessments.

### Sensitivity and robustness analyses

To assess the robustness of the association between hematoma clearance ratio and favorable outcome, we constructed additional multivariable LR models that sequentially adjusted for IVH and onset-to-surgery time.

As summarized in Supplementary Tables 5.1, 5.3, the association between a higher clearance ratio and favorable outcome remained strong and statistically significant across all models for both 6-month and 12-month outcomes. The coefficient and Odds Ratio (OR) for the clearance ratio demonstrated remarkable stability. For the 6-month models, the coefficient varied between 5.40 and 5.60, with a maximum relative change of 2.72%. In the 12-month models, the coefficient varied between 5.89 and 6.15, with a maximum relative change of 4.34%.

The AUC values reported in Supplementary Tables 5.2, 5.4 were derived from alternative adjusted LR sensitivity models and were used to examine whether additional adjustment for IVH and onset-to-surgery time materially changed the model estimates. Moreover, the quantitative validation for excluding GCS in the 6-month model is also confirmed herein, as the model with GCS showed nearly identical performance and minimal impact on the clearance ratio’s association compared to the LASSO-selected model (Supplementary Table 6).

## Discussion

The principal finding of this study is that hematoma clearance ratio, together with age, admission GCS score, and ICH grade, was associated with long-term functional outcomes after hematoma evacuation. In repeated internal validation, LR achieved performance comparable to the evaluated ML models. Given the modest sample size, these findings should be interpreted as exploratory and require external validation.

### Interpretation and exploratory threshold of hematoma clearance ratio

Hematoma clearance ratio was one of the most clinically interpretable variables in the final model. Compared with absolute hematoma volume removed, clearance ratio provides a normalized description of the relative extent of hematoma reduction and may allow comparison across patients with different baseline hematoma volumes (Supplementary Figure 2). This is clinically relevant because two patients may have similar absolute residual volumes or similar absolute volumes removed, but the proportional degree of hematoma reduction may differ substantially depending on the initial hematoma burden. In our cohort, which included a clinically relevant range of initial hematoma volumes, the comparative analysis of clearance ratio and absolute volume removed suggested that clearance ratio provided complementary prognostic information as a relative postoperative hematoma-reduction metric (Supplementary Table 7).

To further explore the relationship between clearance and outcome, we performed a threshold analysis. In the present study, the Youden index suggested a statistical threshold of 67.3%, whereas RCS analysis indicated a candidate cut point near 81% (Supplementary Table 8). These two thresholds should not be interpreted as contradictory; rather, they reflect different statistical criteria and different trade-offs between sensitivity and specificity. The Youden-derived value maximizes the combined sensitivity and specificity, whereas the RCS-derived value reflects a potential change in the shape of the association between clearance ratio and the probability of favorable outcome.

We therefore report the 81% value as an exploratory RCS-derived candidate cut point. A higher clearance ratio is clinically intuitive because greater hematoma reduction is generally desirable after evacuation. This is further supported by the observed trend that a higher hematoma clearance rate is associated with more favorable long-term prognosis at 12 months compared to 6-month outcomes. This finding may help generate a testable hypothesis for future studies, providing neuro-surgeons with a potential postoperative quality indicator or stratification variable. Previous multicenter trials provide clinical context suggesting that the extent of hematoma reduction may be relevant to outcome. The MISTIE III and INVEST trials found that thresholds of ICH removal influenced outcomes similarly across both procedures. Patients had a comparable likelihood of achieving a favorable outcome within a broad therapeutic time window in both trials (25). In contrast, the CLEAR III trial showed that although outcomes did not differ significantly between the two groups, the alteplase group had a lower probability of adverse events (26).

Nevertheless, the interpretation of clearance ratio requires caution. Clearance ratio is mathematically derived from preoperative and postoperative hematoma volumes and should therefore not be regarded as a biological predictor independent of its component volume measures. Instead, it should be understood as a postoperative surgical-efficiency metric that summarizes relative hematoma reduction. Baseline hematoma volume, postoperative residual volume, absolute volume removed, and clearance ratio are mathematically and clinically related. Therefore, our findings do not imply that clearance ratio replaces these traditional volumetric measures. Rather, clearance ratio may serve as a complementary postoperative metric for prognostic assessment and cross-patient comparison. Future studies with larger external cohorts should directly compare the prognostic value of residual hematoma volume, absolute volume removed, baseline hematoma volume, and clearance ratio.

### Biological plausibility of hematoma reduction

The observed association between greater hematoma reduction and better long-term functional recovery is biologically plausible. When cerebral hemorrhage happens, blood escapes from a ruptured blood vessel into the brain tissue, which can increase pressure inside the skull and harm nearby brain cells (27). This process may result in significant neurological impairment. Serious cases of cerebral hemorrhage can cause limb paralysis, loss of speech, coma, and in severe cases, may be fatal (28). Beyond this initial physical damage, a complex inflammatory response is triggered. Activation of astrocytes, microglia, and release of proinflammatory- and damage-associated molecular patterns are recognized pathophysiological processes in ICH, which drives systemic inflammation and mobilizes inflammatory cells to the injured brain (29–31).

From this perspective, more effective hematoma evacuation may improve prognosis through several non-mutually exclusive mechanisms. First, the mechanical decompression effect is the most direct explanation. Rapid hematoma removal effectively lowers intracranial pressure, alleviates compression and displacement of surrounding brain tissue, and thereby salvages ischemic but not yet necrotic neuronal units in the penumbra. Second, the clearance of neurotoxic substances likely plays a critical role. Following ICH, hematoma breakdown products such as hemoglobin (32) and iron ions trigger intense oxidative stress and inflammatory responses, causing ongoing damage to neural cells (33). Additionally, dyshomeostasis of Cl^−^, K^+^, and Na^+^ occurs acutely post ICH and is associated with edema (34). Surgically removing these toxins can mitigate the extent of secondary brain injury. Third, clearance creates a favorable environment for neural repair. An evacuated, low-toxicity local environment may be more conducive to subsequent neuro-regeneration, synaptic remodeling, and functional reorganization mediated by rehabilitation (35, 36). Our data, which showed a slightly stronger dependence of the 12-month outcome on the clearance ratio, indirectly supports the hypothesis that clearance influences the long-term processes of neural repair.

### Comparison between LR and ML models

In anticipation of the potential of complex ML models, we rigorously evaluated four mainstream models (GNB, LDA, RF and XGBoost). The GNB was employed for its strong theoretical foundation in probability-based classification and inherent resistance to over-fitting, a crucial advantage given our modest dataset. LDA was selected as an interpretable linear classifier that performs well with limited samples, providing clear, clinically-applicable decision boundaries based on variable combinations. RF was chosen for its robust ensemble learning approach, which enhances predictive accuracy through bootstrap aggregation, effectively manages missing data, and provides reliable estimates of feature importance, all while maintaining stability with smaller sample sizes. XGBoost was implemented for its optimized gradient-boosting framework, which excels at capturing complex predictor interactions through sequential model building, while employing regularization techniques to minimize overfitting risks. While other advanced machine learning techniques, such as SVM or NN, could have been considered, they were deemed less suitable for our investigation due to their typically greater data requirements compared to the selected models. Recent ICH-related ML studies, including ML-based prediction of spontaneous ICH recurrence, further support the growing role of algorithmic approaches in cerebrovascular risk stratification (12). However, given our modest single-center cohort, we selected LR as the final model for its comparable performance, lower complexity, and greater clinical interpretability.

From a methodological perspective, another noteworthy finding is that in our moderately-sized dataset, the LR model performed on par with, or even surpassed, certain more complex ML algorithms such as Random Forest and XGBoost. This is likely because, after effective variable selection via LASSO, the predictive relationships are essentially approximately linear. Under these conditions, parametric models like LR, with their lower model complexity and variance, demonstrate better generalization ability with limited data and are less prone to overfitting (37). Similar studies can valid this result (37–39). The theoretical advantages of complex ML models are difficult to realize without massive data support. This result strongly suggests that in clinical prediction model research, blindly pursuing model complexity is not optimal; instead, combining clinical knowledge for feature engineering and selecting interpretable models that match the scale of the data often yields greater practical utility.

Although LDA achieved performance similar to LR, we selected LR for its superior clinical interpretability. LDA is useful for classification and can perform well when class separation is approximately linear, but its discriminant coefficients are less commonly interpreted as clinical effect estimates. LR coefficients can be directly exponentiated to odds ratios, clearly quantifying how each predictor—age, GCS score, ICH grade, and hematoma clearance ratio—influences the probability of a favorable outcome. This transparency was essential for our goal of developing a postoperative prognostic calculator rather than a black-box classifier.

### Clinical implications

The findings of this study have direct clinical implications. First, the LR-based prediction model we developed is straightforward (incorporating only 4 variables) and can be easily integrated into clinical workflows for postoperative prognostic estimation, helping physicians and families set realistic expectations. Second, our research provides a solid theoretical foundation and an operational blueprint for future randomized controlled trials (RCTs) targeting hematoma clearance ratio. Future prospective studies may evaluate whether hematoma reduction metrics, including clearance ratio, residual postoperative volume, and absolute volume removed, can serve as postoperative quality indicators or stratification variables.

The web calculator was designed to improve accessibility of the final LR model by translating the regression equation into an interactive interface. However, because the model was developed from a single-center retrospective cohort and has not undergone external or prospective validation, the calculator should be considered a research tool for postoperative prognostic estimation. External validation, calibration assessment in independent cohorts, and prospective evaluation of clinical utility are required before routine clinical use.

In addition, recent evidence on secondary prevention after ICH, such as the association between statin use and reduced ICH recurrence, highlights that postoperative prognostic prediction should be integrated with long-term risk management rather than viewed as a stand-alone tool (40).

### Limitations

Several limitations should be acknowledged. First, this was a single-center retrospective study with a modest final sample size of 185 patients, which was below the originally planned sample size. Although repeated internal validation, training-set–restricted preprocessing, LASSO feature selection, and cross-validated tuning were used to reduce overfitting risk, these procedures cannot eliminate optimism, particularly because the workflow involved repeated data splitting, feature selection, multiple model comparisons, sensitivity analyses, and exploratory threshold assessment. This issue is especially relevant for flexible algorithms such as RF and XGBoost, whose performance may be unstable in limited-sample settings. Therefore, the final LR model should be considered exploratory until externally validated.

Second, generalizability may be limited by differences in patient populations, surgical protocols, imaging assessment, and postoperative management across institutions. Outcome assessment based on follow-up visits and telephone interviews may also introduce follow-up or information bias. In addition, the web calculator was technically checked against the underlying LR equation but has not been prospectively tested or externally validated. Thus, the final LR model, web calculator, and exploratory clearance threshold should be regarded as hypothesis-generating tools for postoperative prognostic estimation rather than established clinical decision-support instruments.

## Conclusion

This single-center retrospective study showed that hematoma clearance ratio was associated with long-term functional outcome after stereotactic hematoma evacuation for ICH. As an intuitive postoperative volumetric metric, clearance ratio may help neurosurgeons summarize the extent of hematoma reduction after CT assessment and support postoperative prognostic estimation, rehabilitation planning, and communication with patients and families. The LR model showed internally validated performance comparable to the evaluated ML models and was selected for its interpretability. The candidate threshold near 81% remains exploratory and requires external validation before being used as a clinical or operative target.

## Data Availability

The dataset is not publicly available due to patient privacy concerns. Access is only possible upon reasonable request to the corresponding authors. Requests to access the datasets should be directed to Hangzhe Sun, hangzhesun0823@16.com.

## Footnotes

1
https://ichprediction.shinyapps.io/shiny_app/
