# Supplementary material for: Predicting long-term outcomes in intracerebral hemorrhage: a comparative study of machine learning models highlights the prognostic value of hematoma clearance
Source: Front Neurol. 2026 Aug 28;17:1828200. doi: 10.3389/fneur.2026.1828200 (PMC13564256; doi:10.3389/fneur.2026.1828200)
Supplement: Supplementary file 1 [file Supplementary_file_1.docx]

**Supplementary Table 1. Determination of modified Rankin Scale (mRS) Scoring Scale.**

| **Patient Condition** | **Score** |
| --- | --- |
| No symptoms at all | 0 |
| No significant disability despite symptoms  Able to perform all usual duties and activities | 1 |
| Slight disability  Unable to perform all previous activities  able to look after own affairs without assistance | 2 |
| Moderate disability  Require some help  Able to walk without assistance | 3 |
| Moderately severe disability  Unable to walk without assistance  Unable to attend to own bodily needs without help | 4 |
| Severe disability  Bedridden  Incontinent  Require constant nursing care and attention | 5 |
| Death | 6 |

**Supplementary Table 2. Determination of** **intracerebral hemorrhage (ICH) Grade.**

| **Component** | **ICH Score Points** |
| --- | --- |
| GCS score |  |
| 3-4 | 2 |
| 5-12 | 1 |
| 13-15 | 0 |
| ICH volume, cm^3^ |  |
| ≥30 | 1 |
| ＜30 | 0 |
| IVH |  |
| Yes | 1 |
| No | 0 |
| Infratentorial origin of ICH |  |
| Yes | 1 |
| No | 0 |
| Age, year |  |
| ≥80 | 1 |
| ＜80 | 0 |
|  |  |
| **Total ICH Score** | 0-6 |

Note: Glasgow Coma Scale (GCS) score indicates GCS score on initial presentation (or after resuscitation); ICH volume, volume on initial Computed Tomography (CT) calculated using ABC/2 method; Intraventricular hemorrhage (IVH), presence of any IVH on initial CT.

| **Behavior** | **Response** | **Score** |
| --- | --- | --- |
| Eye opening response (E) | Spontaneously | 4 |
|  | To speech | 3 |
|  | To pain | 2 |
|  | No response | 1 |
| Best verbal response (V) | Oriented to time, place and person | 5 |
|  | Confused | 4 |
|  | Inappropriate words | 3 |
|  | Incomprehensible sounds | 2 |
|  | No response | 1 |
| Best motor response (M) | Obey commands | 6 |
|  | Moves to localized pain | 5 |
|  | Flexion withdrawal from pain | 4 |
|  | Abnormal flexion | 3 |
|  | Abnormal extension | 2 |
|  | No response | 1 |
|  | | |
| **Total score** | Best response | 15 |
|  | Comatose client | 8 or less |
|  | Totally unresponsive | 3 |

**Supplementary Table 3. Determination of Glasgow Coma Scale (GCS).**

Note: The GCS score (range 3-15) is the sum of the best responses in the Eye (E), Verbal (V), and Motor (M) categories. A lower score indicates a deeper level of coma: mild (13-14), moderate (9-12), and severe (3-8). A score of 2T is assigned to intubated/tracheostomy patients in deep coma who cannot vocalize. The motor score is assessed for each side, and the higher value is used for the total GCS. The modified GCS should record both the best/worst response and the left/right motor scores.

**Supplementary Table 4. Baseline Characteristics of the Training and Validation Cohorts.**

| **Characteristic** | **Testing Cohort**  N = 56 | **Training Cohort**  N = 129 | **p-value**^1^ | **Overall**  N = 185 |
| --- | --- | --- | --- | --- |
| **Gender (Female)** |  |  | 0.6 |  |
| Male | 40 (71%) | 98 (76%) |  | 138 (75%) |
| Female | 16 (29%) | 31 (24%) |  | 47 (25%) |
| **Age (years)** | 56 ± 15 | 58 ± 13 | 0.4 | 57 (47, 68) |
| **BMI** | 24.6 (21.7, 27.9) | 23.9 (21.5, 26.4) | 0.3 | 24.0 (21.5, 26.7) |
| **GCS** | 10.5 (7.0, 13.0) | 11.0 (8.0, 13.0) | 0.4 | 11.0 (8.0, 13.0) |
| **ICH Grade** |  |  | 0.2 |  |
| 0 | 1 (1.8%) | 9 (7.0%) |  | 10 (5.4%) |
| 1 | 13 (23%) | 35 (27%) |  | 48 (26%) |
| 2 | 21 (38%) | 56 (43%) |  | 77 (42%) |
| 3 | 15 (27%) | 23 (18%) |  | 38 (21%) |
| 4 | 6 (11%) | 5 (3.9%) |  | 11 (5.9%) |
| 5 | 0 (0%) | 1 (0.8%) |  | 1 (0.5%) |
| **Alcohol Use** | 23 (41%) | 51 (40%) | >0.9 | 74 (40%) |
| **Smoke (>3 years)** | 24 (43%) | 55 (43%) | >0.9 | 79 (43%) |
| **Hypertension** | 33 (59%) | 84 (65%) | 0.5 | 117 (63%) |
| **Diabetes** | 8 (14%) | 8 (6.2%) | 0.13 | 16 (8.6%) |
| **Anticoagulant Use** |  |  | >0.9 |  |
| None | 50 (89%) | 116 (90%) |  | 166 (90%) |
| Antiplatelet | 3 (5.4%) | 7 (5.4%) |  | 10 (5.4%) |
| Anticoagulant | 3 (5.4%) | 6 (4.7%) |  | 9 (4.9%) |
| **Hemorrhage Location** |  |  | 0.8 |  |
| Deep | 46 (82%) | 111 (86%) |  | 157 (85%) |
| Lobar | 5 (8.9%) | 10 (7.8%) |  | 15 (8.1%) |
| Both | 5 (8.9%) | 8 (6.2%) |  | 13 (7.0%) |
| **Preoperative Hematoma Volume** | 45 (31, 61) | 42 (29, 57) | 0.7 | 42 (30, 59) |
| **Admission SBP (mmHg)** | 153 (136, 171) | 160 (139, 180) | 0.3 | 157 (138, 179) |
| **Midline Shift** | 49 (88%) | 103 (80%) | 0.3 | 152 (82%) |
| **Spot Sign** | 3 (5.4%) | 4 (3.1%) | 0.7 | 7 (3.8%) |
| **IVH** | 27 (49%) | 52 (40%) | 0.3 | 79 (43%) |
| **Surgical Device** |  |  | **0.044** |  |
| Frame | 11 (20%) | 35 (27%) |  | 46 (25%) |
| Brainlab | 28 (50%) | 75 (58%) |  | 103 (56%) |
| Remebot | 17 (30%) | 19 (15%) |  | 36 (19%) |
| **Onset-to-surgery Time** | 49 (33, 90) | 50 (27, 79) | 0.4 | 50 (30, 82) |
| **Procedure Duration** | 51 (40, 60) | 55 (45, 65) | 0.15 | 51 (45, 60) |
| **Postoperative Hematoma Volume** | 13 (7, 23) | 10 (5, 18) | **0.018** | 11 (5, 22) |
| **Clearance Ratio** | 0.60 (0.52, 0.77) | 0.75 (0.61, 0.85) | **0.002** | 0.73 (0.56, 0.85) |
|  | | | | |
| 1: T test (Age); Mann-Whitney U test (Other continuous); Chi-square test (All categorical). Note: Body Mass Index (BMI); Glasgow Coma Scale (GCS); Intracerebral Hemorrhage (ICH); Systolic Blood Pressure (SBP). | | | | |

**Supplementary Table 5.1. Sensitivity Analysis of Hematoma Clearance Ratio Association with 6-Month Outcome Across Different Multivariable Models.**

| **Model** | **Clearance Ratio Coefficient** | **Clearance Ratio OR** | **Clearance Ratio p** | **Relative Change** |
| --- | --- | --- | --- | --- |
| Original | 5.45 | 232.92 | 1.21e-06 | 0.00 |
| With IVH | 5.40 | 222.17 | 1.69e-06 | 0.87 |
| With  Onset-to-surgery Time | 5.60 | 270.18 | 9.18e-07 | 2.72 |

Note: Original Model: Age + GCS score + ICH grade + Clearance Ratio; With IVH Model: Age + GCS score + ICH grade + Clearance Ratio + IVH; With Onset-to-surgery Time Model: Age + GCS score + ICH grade + Clearance Ratio + Onset-to-surgery Time.

**Supplementary Table 5.2. Secondary adjusted sensitivity analysis for the 6-month LR model**

| **Model** | **AIC** | **BIC** | **AUC** | **Accuracy** | **Sensitivity** | **Specificity** |
| --- | --- | --- | --- | --- | --- | --- |
| Original | 206.96 | 223.06 | 0.80 | 0.76 | 0.81 | 0.71 |
| With IVH | 208.13 | 227.42 | 0.80 | 0.75 | 0.81 | 0.70 |
| With  Onset-to-surgery Time | 207.99 | 227.31 | 0.80 | 0.76 | 0.83 | 0.69 |

Note: AIC: Akaike Information Criterion; BIC: Bayesian Information Criterion; AUC: Area Under Curve.

**Supplementary Table 5.3. Sensitivity Analysis of Hematoma Clearance Ratio Association with 12-Month Outcome Across Different Multivariable Models.**

| **Model** | **Clearance Ratio** **Coefficient** | **Clearance Ratio OR** | **Clearance Ratio p** | **Relative Change** |
| --- | --- | --- | --- | --- |
| Original | 5.89 | 362.82 | 6.28e-07 | 0.00 |
| With IVH | 5.91 | 367.52 | 7.07e-07 | 0.22 |
| With  Onset-to-surgery Time | 6.15 | 468.58 | 4.09e-07 | 4.34 |

**Supplementary Table 5.4. Secondary adjusted sensitivity analysis for the 12-month LR model.**

| **Model** | **AIC** | **BIC** | **AUC** | **Accuracy** | **Sensitivity** | **Specificity** |
| --- | --- | --- | --- | --- | --- | --- |
| Original | 184.69 | 200.79 | 0.84 | 0.76 | 0.90 | 0.60 |
| With IVH | 185.53 | 204.82 | 0.84 | 0.76 | 0.90 | 0.59 |
| With  Onset-to-surgery Time | 184.68 | 204.00 | 0.85 | 0.75 | 0.89 | 0.59 |

**Supplementary Table 6.1. Sensitivity analysis of the hematoma clearance ratio’s association with 6-month outcome across different multivariable Logistic Regression models.**

| **Model** | **Clearance Ratio**  **Coefficient** | **Clearance Ratio**  **OR** | **Clearance Ratio**  **p** | **Relative**  **Change** |
| --- | --- | --- | --- | --- |
| Original | 5.54 | 253.61 | 7.60e-07 | 0.00 |
| With  GCS | 5.45 | 232.92 | 1.22e-07 | 1.54 |

Note: Original Model: Age + ICH Grade + Clearance Ratio; With GCS Model: Age + ICH Grade + Clearance Ratio + GCS score.

**Supplementary Table 6.2. Comparative performance metrics of the 12-month prognostic Logistic Regression models.**

| **Model** | **AIC** | **BIC** | **AUC** | **Accuracy** | **Sensitivity** | **Specificity** |
| --- | --- | --- | --- | --- | --- | --- |
| Original | 205.73 | 218.61 | 0.80 | 0.75 | 0.80 | 0.70 |
| With  GCS | 206.96 | 223.06 | 0.80 | 0.76 | 0.81 | 0.71 |

**Supplementary Table 7.1. Predictive Performance of Clearance Ratio and Volume Removed (6-month).**

| **Model** | **AUC** | **Sensitivity** | **Specificity** | **Accuracy** | **PPV** | **NPV** |
| --- | --- | --- | --- | --- | --- | --- |
| Clearance Ratio | 0.80 | 0.74 | 0.79 | 0.77 | 0.75 | 0.78 |
| Volume Removed | 0.72 | 0.79 | 0.57 | 0.65 | 0.60 | 0.73 |

Note: Clearance Ratio Model: Age + GCS score + ICH grade + Clearance Ratio; Volume Removed Model: Age + GCS score + ICH grade + Volume Removed. PPV: Positive Predictive Value; NPV: Negative Predictive Value.

**Supplementary Table 7.2. Predictive Performance of Clearance Ratio and Volume Removed (12-month).**

| **Model** | **AUC** | **Sensitivity** | **Specificity** | **Accuracy** | **PPV** | **NPV** |
| --- | --- | --- | --- | --- | --- | --- |
| Clearance Ratio | 0.84 | 0.75 | 0.85 | 0.81 | 0.76 | 0.84 |
| Volume Removed | 0.77 | 0.82 | 0.63 | 0.70 | 0.58 | 0.85 |

**Supplementary Table 8. Performance Comparison of Two Hematoma Clearance Ratio Thresholds.**

| **Threshold** | **Sensitivity** | **Specificity** | **Accuracy** | **PPV** | **NPV** |
| --- | --- | --- | --- | --- | --- |
| 67.3% (Youden) | 0.770 | 0.593 | 0.692 | 0.688 | 0.699 |
| 81.0% (RCS) | 0.465 | 0.744 | 0.595 | 0.676 | 0.547 |

Note: RCS: Restricted Cubic Spline.

**Supplementary Figure 1. Repeated resampling framework.**

**Supplementary Figure 2. Distribution of Initial Hematoma Volumes.**
